# Concomitant coexistence of ACTH‐dependent and independent Cushing syndrome

**DOI:** 10.1002/ccr3.5834

**Published:** 2022-05-09

**Authors:** Ach Taieb, Saad Ghada, Gorchène Asma, Ben Abdelkrim Asma, Kacem Maha, Ach Koussay

**Affiliations:** ^1^ Department of Endocrinology University Hospital of Farhat Hached Sousse Tunisia; ^2^ Faculty of Medicine Ibn El Jazzar University of Sousse Sousse Tunisia

**Keywords:** adrenal adenomas, cortisol, Cushing disease, transsphenoidal surgery

## Abstract

We report a case of a patient who presented for ACTH‐dependent Cushing after a confirmed hypercortisolism and an inadequate normal ACTH. A transsphenoidal surgery of a pituitary picoadenoma has been done. After surgery, the patient showed the persistence of hypercortisolism. CT scan revealed adrenal adenomas removed surgically and improved the patient.

## Abstract 2

The concomitant coexistence of ACTH‐dependent and independent Cushing syndrome remains extremely rare. The difficulty is to determine whether the adrenal dependency existed at the time of the diagnosis or if it developed postoperatively.

## 1 INTRODUCTION

Cushing syndrome (CS) is a rare condition with a prevalence of 10–15 per million people.^1^ It is the result of excess cortisol levels. This exposure to excess glucocorticoids leads to several complications like cardiovascular, metabolic, and skeletal diseases, not to mention psychiatric disturbances. The hypercortisolism can be due to overproduction by the adrenal glands or due to the overproduction of adrenocorticotropic hormone (ACTH) by the pituitary gland. Identifying the causal lesion can be straightforward based on the laboratory test and imaging but can be very often challenging. The two most common aetiologies of ACTH‐independent CS are adrenal adenoma and carcinoma. Adrenal adenomas are responsible for approximately 10–15% of cases of CS, and adrenal carcinomas are responsible for <5% of cases of CS.^2^ In ACTH‐dependent CS, the adrenal glands would be expected to develop diffuse hyperplasia and to appear uniformly enlarged in imaging studies. Although this is usually the case, macronodular hyperplasia is present in 10–15% of patients with Cushing disease (CD).^3^ Nodules are generally bilateral, but when macronodular hyperplasia involves a single gland, it could be confused with an adrenal adenoma. However, the coexistence of cortisol‐secreting adrenal adenomas with a CD has been described in only a few case reports.^3^ We report a case of a 46‐year‐old woman who presented with both CD and CS.

## 2 CASE PRESENTATION

**FIGURE 1:**
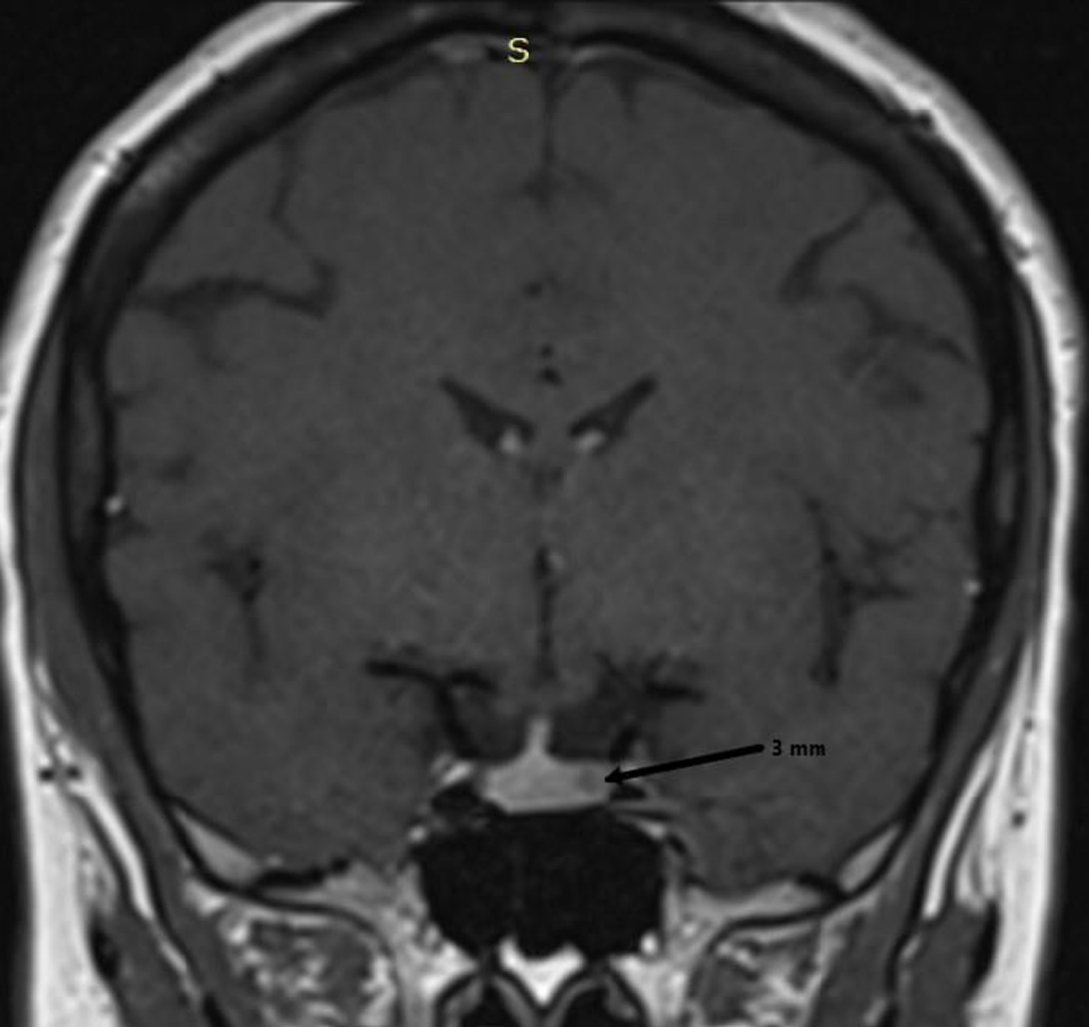
Pituitary MRI with gadolinium contrast, coronal T1‐weighted image showing a 3‐mm picoadenoma on the left side of the pituitary.

The patient is a 46‐year‐old woman who was admitted to our endocrinology department for exploration of classic signs of hypercortisolism, which had developed over a period of 3 years. Her medical history included recently diagnosed arterial hypertension and diabetes. She was on antihypertensive (captopril 25 1 pillx2 and amlodipine) and antidiabetic drugs (metformin 850g 1pillx2). Her clinical examination showed facial plethora, easy bruising, and hirsutism (Table 1). Her initial hormonal tests showed unsuppressed cortisol at 230 ng/ml and high urine‐free cortisol (UFC) at 30 µg/24 h after the 2‐mg 2‐day dexamethasone suppression test. Two measures of ACTH were inadequately normal at 19 and 21 ng/L, respectively. An ACTH‐dependent CD was thus diagnosed. An 8‐mg dexamethasone suppression test showed more than a 50% decrease in cortisol levels in favor of a CD. A pituitary MRI showed a 3‐mm picoadenoma on the left side of her pituitary (Figure 1).

**TABLE 1:** Summary of clinical and biochemical parameters before and after surgery

	Before surgery	Three months after surgery
Personal history	Diabetes, hypertension	Persistent
Clinical signs	Catabolic symptoms: Facial plethora, hirsutism	Persistent
BMI (kg/m²)	31.2	29
Arterial pressure (mmHg)	Systolic [15–16]; diastolic [9–10]	systolic [14–17]; diastolic [9–10]
HBA1C (%)	7	8,3
Bone densitometry	Femoral osteopenia	Femoral osteopenia
Calcemia(mmol/L)	2.3	2.25
Kalemia (mmol/L)	4	3.4

**FIGURE 2:**
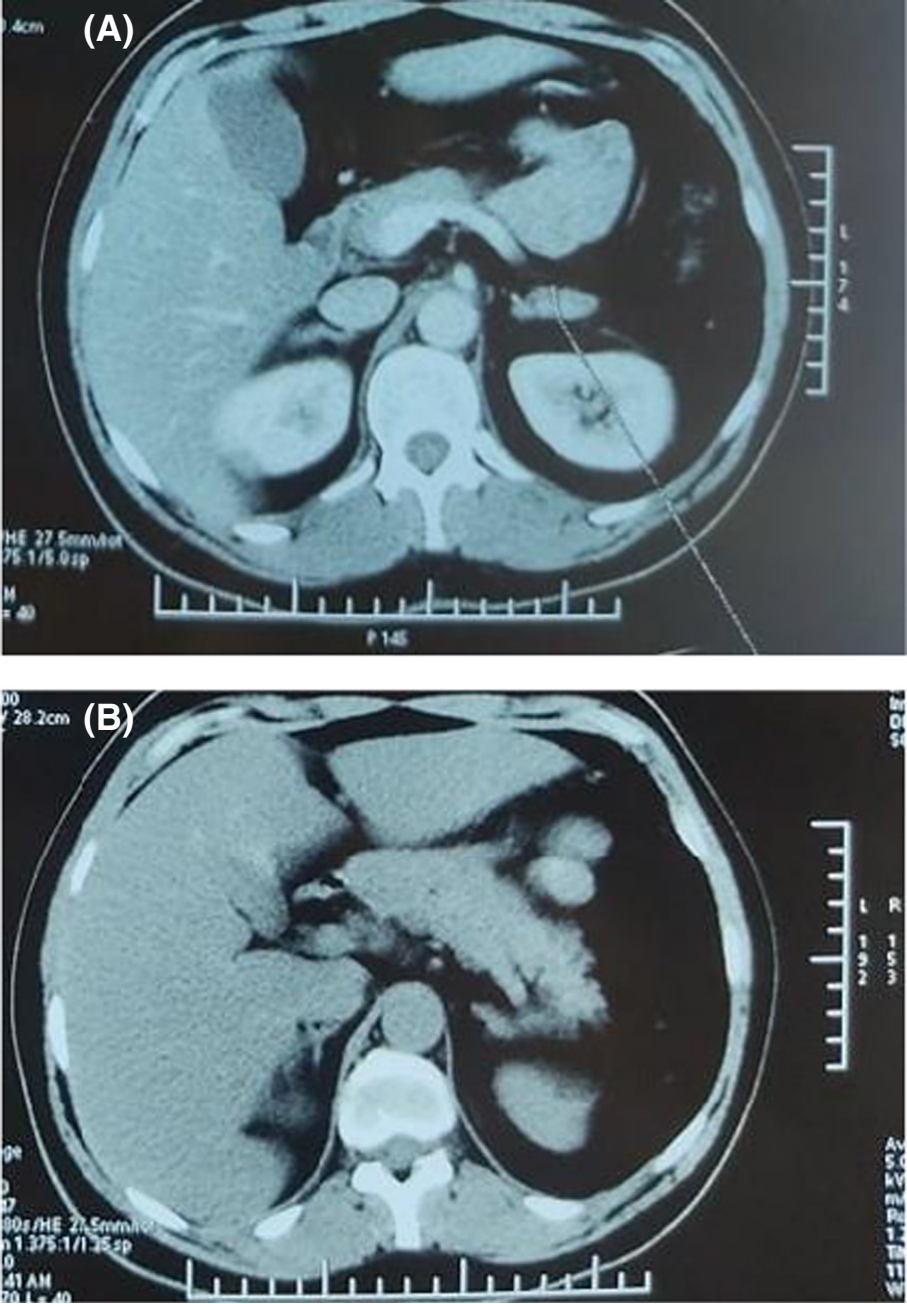
(A, B) CT scan of the abdomen demonstrating two adenomas in the left and right adrenal glands, measuring 30 × 17 mm and 15 × 9 mm, respectively.

The patient was referred to a neurosurgeon, and a transsphenoidal pituitary adenomectomy was performed. Immunohistochemical staining was exclusively positive for ACTH. The pathologist's diagnosis was a corticotroph tumor. Ki‐67 was estimated at 2%. Three months after the surgery, the patient still had not noticed any improvement. She was admitted to our department for postoperative assessment. Her physical examination did not show any improvement. Indeed, she still had physical signs of hypercortisolism. Her metabolic and heart pressure features did not improve (Table 1). The laboratory tests confirmed the lack of biochemical remission since her 2‐mg 2‐day DST showed high cortisol at 105 ng/ml and urinary free cortisol remained high. (Table 2). A 3‐month postoperative pituitary MRI scan did not show a remnant pituitary adenoma. Her ACTH decreased to 6 ng/L, which was in favor of a successful surgery. An adrenal computed tomography scan was performed and revealed two adenomas in the right and left adrenal glands, which were both rich in lipids with a 7 HU density. The left and right adenomas measured 30 × 17 mm and 15 × 9 mm, respectively (Figure 2A,B). Based on a suppressed level of ACTH and adrenal adenomas, we concluded at this point to an ACTH‐independent CS. The patient was referred later to a urologist and underwent bilateral adrenalectomy. Her symptoms improved remarkably. She developed an adrenal insufficiency and was put on hydrocortisone.

**TABLE 2:** Summary of hormonal assessment before and after surgery

	Before surgery	Three months after surgery
Cortisol after 2‐mg 2‐day DST (nmol/dl)	634.48	289.65
Urinary free cortisol after 2‐mg 2‐day DST (µg/24 h)	30	25
Cortisol after 8‐mg dexamethasone suppression test	Cortisol's decrease of 50%	_
ACTH (pg/ml)	21	4
TSH (mUI/L) / FT4 (pmol/L)	2.5 / 13	1.94/5.8
Prolactin (mUI/ml)	715	225
Testosterone (ng/ml)	0.52	0.49

## 3 DISCUSSION

Here, we present a patient with an unusual coexistence of a confirmed CD and a cortisol‐secreting adrenal adenoma. CD is the most common cause of CS.^4^ It results from an ACTH‐secreting pituitary tumor. First‐line treatment is transsphenoidal pituitary surgery with a success rate estimated at 70% depending on the surgeon's experience mainly.^5^ Failed surgery is, however, reported in 7–30%.^5^ It is usually due to a residual invasive tumor or in rare cases an incorrect diagnosis mainly an ectopic ACTH hypersecretion or corticorph hyperplasia. Our patient had predictive factors of a potentially successful surgery, including a visualized and infracentimetric size tumor on MRI.^4^ The low ACTH level postsurgery, the positive immunohistochemical staining, and the absence of remnant tumor on the MRI were all in favor of a successful surgery. However, the patient showed no clinical or biological remission. The differential diagnosis of a patient with overt hypercortisolism, measurable ACTH levels, and an adrenal nodule should include the coexistence of pituitary or ectopic CS and a nonfunctioning adrenal adenoma, ectopic ACTH, and/or CRH secretion by an adrenal nodule, and as focused herein, an adrenal adenomas. ACTH levels were low at 6 ng/L, and computed adrenal tomography showed surprisingly two bilateral adrenal adenomas. These findings matched well with an ACTH‐independent CS.

According to Albiger et al.,^6^ adrenal nodules in CD are more common than what was previously thought with an estimated 37% prevalence. This is particularly more frequent in older patients with long‐standing diseases. This is explained by a long exposure to ACTH that stimulates cell proliferation leading to nodules and hyperplasia. These nodules usually shrink after disease remission.^7^ However, few case reports showcased the autonomy of secretion of these adrenal glands like the case of our patient. A case report by Hocher et al.^8^ described a transition from pituitary dependency to adrenal dependency. ACTH measurement using a sensitive ACTH assay is a fundamental step in the diagnosis but difficulties could arise when long‐standing ACTH hypersecretion has led to adrenal nodule formation. Indeed, these nodules may eventually develop a variable degree of autonomy with heterogeneous results of ACTH measurement and dynamic testing. The possible transition from a previous ACTH‐dependent to a later adrenal‐dependent phase is supported by the in vitro findings of Lamberts et al.^9^ who studied the ACTH responsiveness of adrenal cells from unilateral adrenal nodules removed in patients with pituitary‐dependent CS. It seems difficult in hindsight to determine whether the adrenal dependency existed at the time of diagnosis or if it developed postoperatively.^10^ Perhaps, the inadequately normal low ACTH levels before surgery should have drawn attention to a possible suppression by the already autonomous adrenal adenomas. Morphological imaging modalities, including CT and magnetic resonance imaging, are not useful for distinguishing between a hormonally hyperactive and nonfunctioning adrenal mass, whereas both 131I‐NP‐59 scintigraphy and adrenal venous sampling are reportedly useful for identifying adrenal CS.^2^ Only a few studies have reported the use of adrenal venous sampling for CS with bilateral masses, of which the most well‐known was conducted by the Mayo Clinic.^11^ They reported that catheterization can be considered successful if the serum adrenaline level in the adrenal vein exceeds that in the peripheral vein by >100 pg/ml. This procedure has some limitations, including difficulty approaching a small right adrenal vein and the unavailability of this examination in all medical structures as it was our case.^1^

## 4 CONCLUSION

This case illustrates the unusual coexistence of a CD and a CS. The coexistence of bilateral adrenal masses and CD is documented in only a few reports, which makes this diagnosis very uncommon. After pituitary surgery for a CD, the diagnosis of a patient with overt hypercortisolism should include pituitary recidivism or an adrenal CS. The level of ACTH could indicate the location of the lesion, therefore. CS with bilateral adrenal tumors, both adrenal vein sampling and scintigraphy, should be used to identify the location. However, either examination can provide sufficient information if only one is available.

## AUTHOR CONTRIBUTIONS

Dr Ach Taieb wrote the manuscript. All authors read and approved it.

## CONFLICT OF INTEREST

The authors declare that there is no conflict of interest that could be perceived as prejudicing the impartiality of the research reported.

## CONSENT

Written and informed consent was obtained from the patient for publication of the submitted article.
